# Genetic Correlation and Mendelian Randomization Analyses Support Causal Relationships Between Instant Coffee and Age‐Related Macular Degeneration

**DOI:** 10.1002/fsn3.70439

**Published:** 2025-06-14

**Authors:** Qi Jia, Zhijian Zha, Si Li, Yong Zhang, Lan Ke, Siwei Liu

**Affiliations:** ^1^ Department of Ophthalmology Shiyan Taihe Hospital, Hubei University of Medicine Shiyan Hubei China; ^2^ College of Traditional Chinese Medicine Hubei University of Chinese Medicine Wuhan Hubei China; ^3^ Department of Epidemiology and Biostatistics School of Public Health, Tongji Medical College, Huazhong University of Science and Technology Wuhan Hubei China

**Keywords:** age‐related macular degeneration, Bayesian colocalization, coffee, genetic association, genetic correlation, Mendelian randomization

## Abstract

Coffee is a popular beverage, and previous cohort studies suggest it may reduce the risk of age‐related macular degeneration (AMD). However, confounding factors in these studies necessitate further exploration of causal relationships using advanced methods. We obtained data on coffee consumption from genome‐wide association studies (GWAS) and the latest AMD‐related GWAS summary data from the Finngen consortium R11. We assessed their genetic correlation using linkage disequilibrium score regression (LDSC), explored causal associations using Mendelian randomization (MR), and identified shared genetic loci via colocalization. Our results revealed a genetic correlation between instant coffee consumption and dry AMD, with each standard deviation (SD) increase in instant coffee intake associated with a corresponding odds ratio (OR) of approximately 6.92 for dry AMD, indicating a 6.92‐fold increased risk. However, colocalization analysis did not show shared genetic variants between instant coffee consumption and AMD. Instant coffee may increase the risk of AMD, and reducing its intake could help prevent dry AMD. People at high‐risk for AMD should avoid instant coffee. This study aids clinicians in identifying dietary factors, particularly instant coffee consumption, as potential risks for AMD. By providing genetically based causal evidence, our findings support the development of personalized AMD prevention strategies. Clinicians can advise patients to reduce instant coffee intake based on genetic risk profiles, offering a precision approach to reduce dry AMD risk. These interventions may significantly contribute to AMD prevention and treatment.

## Graphical abstract

Our results revealed a genetic correlation between instant coffee consumption and dry AMD, with each standard deviation (SD) increase in instant coffee intake associated with a corresponding odds ratio (OR) of approximately 6.92 for dry AMD, indicating a 6.92‐fold increased risk. Instant coffee may increase the risk of AMD, and reducing its intake could help prevent dry AMD. People at high‐risk for AMD should avoid instant coffee.

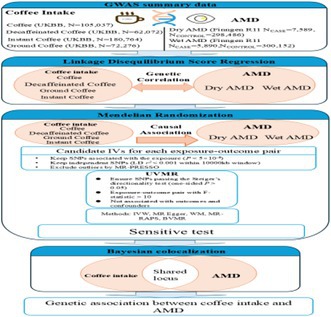

## 1 Introduction

Age‐related macular degeneration (AMD) is a common eye disease and one of the leading causes of irreversible blindness. AMD can be classified into two types: wet AMD and dry AMD, distinguished by the presence or absence of choroidal neovascularization (van Lookeren Campagne et al. 2014). Epidemiological studies indicate that AMD affects nearly 200 million people worldwide, with the number expected to reach 290 million by 2040 (Fleckenstein et al. 2024). Research has shown that genetic factors play a major role in the development of AMD, with many AMD‐related genetic loci, susceptibility genes, and risk factors having been identified, providing important clues for studying the mechanisms of AMD (Klein et al. 2005; Fritsche et al. 2014; Stahl 2020). However, given the unclear disease mechanisms and the complexity of treatment, slowing disease progression and timely prevention are particularly important (Deng et al. 2022).

Coffee is a common beverage, and the association between coffee consumption and human health has been widely studied. The effects of coffee on eye diseases and aging have been previously demonstrated (Kim et al. 2021; Li et al. 2022; Wei et al. 2023). However, only a few studies have researched coffee and AMD; previous cohort studies have shown that coffee can reduce the risk of AMD and slow its progression (Chiu et al. 2017). Besides, cohort studies are often subject to biases due to confounding factors and reverse causation, leading to errors in the results (Song and Chung 2010; Vassy et al. 2018). Therefore, it is necessary to use new methods to comprehensively assess the genetic association between coffee consumption and AMD.

Linkage disequilibrium score regression (LDSC) can evaluate genetic correlation using GWAS summary statistics, and this method is typically unaffected by sample overlap (Bulik‐Sullivan et al. 2015; Zhang et al. 2021). Mendelian randomization (MR) is a method that uses summary data from genome‐wide association studies (GWAS) to assess the causal effects of diseases, diet, and other factors on disease outcomes (Birney 2022; Sanderson et al. 2022). MR controls for potential confounding factors using instrumental variables (IVs) and is not affected by environmental confounding and reverse causation (Sekula et al. 2016). Bayesian colocalization is a method used to determine whether two or more traits or diseases share the same genetic variants, with the aim of exploring potential causal variants between traits (Rasooly et al. 2022). Combining the results of these three methods can provide more reliable evidence of the genetic variation and causal association between coffee consumption and AMD (Liu et al. 2024).

In this study, we performed causal inference on the relationship between coffee consumption and AMD risk. We integrated genetic correlation, MR, and Bayesian colocalization methods to analyze their genetic association, aiming to explore the impact of coffee consumption on AMD risk and their genetic relationship.

## 2 Materials and Methods

### 2.1 Study Design

Firstly, we obtained summary‐level GWAS data from publicly available databases. We then analyzed the genetic correlation between different types of coffee consumption and AMD using LDSC. Next, we assessed the causal relationship between coffee consumption and AMD risk using univariable Mendelian randomization (UVMR), employing a stringent data selection process and combining five MR methods to reveal the causal association, with sensitivity analyses enhancing the credibility of the findings. Finally, we explored shared genetic variants between different types of coffee consumption and AMD using Bayesian colocalization. By integrating these three approaches, we aimed to draw reliable conclusions regarding the association between various types of coffee consumption and AMD risk.

### 2.2 Data Sources

#### 2.2.1 Coffee Consumption GWAS Data

For coffee consumption data, we used summary statistics from the GWAS on food consumption by Pirastu et al. (2022) which has detailed descriptions of the inclusion criteria for coffee. Following previous methods (Li et al. 2022), we equated coffee consumption with the amount of coffee intake. The GWAS summary data included for coffee consumption were total coffee consumption (*N* = 105,037), decaffeinated coffee consumption (*N* = 62,072), ground coffee intake (*N* = 72,276), and instant coffee consumption (*N* = 180,764) (Table 1).

**TABLE 1:** The source of GWAS data.

Traits	Number of case	Number of control	Total number	Data source
Coffee consumption	NA	NA	806,834	UK biobank
Decaffeinated coffee consumption	NA	NA	62,072	UK biobank
Ground coffee consumption	NA	NA	72,276	UK biobank
Instant coffee consumption	NA	NA	180,764	UK biobank
Wet AMD	5890	300,152	306,042	Finngen
Dry AMD	7589	298,486	306,075	Finngen

Abbreviation: AMD, Age‐related Macular Degeneration.

#### 2.2.2 AMD GWAS Data

We obtained GWAS datasets for dry AMD (*N*_CASE_ = 7589, *N*_CONTROL_ = 298,486) and wet AMD (*N*_CASE_ = 5890, *N*_CONTROL_ = 300,152) from publicly available data released by the Finngen consortium (Kurki et al. 2023). For dry AMD, cases included individuals aged 50 or older diagnosed by a physician. Similarly, the inclusion criteria for wet AMD cases were individuals aged 50 or older with a physician‐confirmed diagnosis.

### 2.3 Data Analysis

#### 2.3.1 Genome‐Wide Genetic Correlation

LDSC was employed to assess the genetic correlation between coffee consumption and AMD. SNPs with a minor allele frequency (MAF) ≤ 0.01 were excluded, and the remaining SNPs were used for further analysis. Additionally, we conducted bivariate LDSC without constraining the intercept to estimate the genetic correlation (*r*_g_) between coffee consumption and AMD. A BH‐corrected *p*adj < 0.05 was considered indicative of significant correlation. Default parameters were used to calculate the reference data based on the European population from the 1000 Genomes Project (Auton et al. 2015).

#### 2.3.2 Mendelian Randomization (MR) Analysis

In this study, we primarily used univariable Mendelian randomization (UVMR) to select appropriate instrumental variables (IVs) from the SNPs in the exposure traits (Burgess et al. 2017). We selected IVs with *p* < 5 × 10^−8^ as the threshold. Next, we used the 1000 Genomes Project European sample data as the reference panel, retaining SNPs with R (Fleckenstein et al. 2024) < 0.001 and a window size of 10,000 kb to eliminate linkage disequilibrium (Auton et al. 2015). Additionally, palindromic SNPs were excluded. Finally, we used Steiger's Test and MR‐PRESSO to exclude SNPs with directional errors and outliers. The processed GWAS data were then used for subsequent UVMR analysis.

Five MR methods were used to estimate causal effects, with inverse variance weighted (IVW) or Wald ratio models serving as the primary methods for causal relationship assessment. When the number of IVs was ≥ 2, IVW was used as the main method. In addition, MR‐Egger, Bayesian weighted MR (BWMR), weighted median (WM), and MR‐robust adjusted profile score (MR‐RAPS) methods were used to enhance the credibility of the results (Bowden et al. 2016; Zhao, Ming, et al. 2020; Zhao, Wang, et al. 2020). Ideally, all SNPs should be valid, without horizontal pleiotropy affecting the outcome through multiple pathways involving genetic variants. Under such conditions, IVW results are considered reliable. In cases of pleiotropy, MR‐Egger regression was used to provide estimates, with MR‐Egger results given additional weight to reduce the contribution of outliers (Burgess et al. 2016). Cochran's *Q* test was used to assess heterogeneity caused by shared biological pathways involving unmeasured confounders related to exposure and outcome, and when heterogeneity was present, the results of random‐effects IVW were considered reliable (Xiao et al. 2022). The *F* statistic (*F* = beta^2^/se^2^) was used to estimate the strength of the IVs; if the *F* statistic > 10, the IVs were considered to have sufficiently strong correlation with the exposure to ensure that the MR results were not biased by weak instruments (Burgess and Thompson 2011).

#### 2.3.3 Bayesian Colocalization Analysis

To test whether the exposure and outcome share causal variants in the same genomic region, we used colocalization analysis to evaluate five mutually exclusive hypotheses: (1) neither trait has a causal variant (H0); (2) only trait 1 has a causal variant (H1); (3) only trait 2 has a causal variant (H2); (4) each trait has different causal variants (H3); (5) both traits share the same causal variant (H4). Posterior probabilities were used to quantify the evidence for each hypothesis, expressed as PPH0, PPH1, PPH2, PPH3, and PPH4 (Giambartolomei et al. 2018). We selected significant independent SNPs (LD *r* ^2^ = 0.001, *p* < 5 × 10^−8^) from each pair of exposure and outcome in the MR, set a 500 kb upstream and downstream window for analysis, and calculated the final colocalization result as the average PPH4 across all regions. A PPH4 > 0.75 suggested a possible shared genetic variant in the region between coffee consumption and AMD risk.

### 2.4 Statistical Analysis

All MR analyses were conducted using R (version 4.4.1).

## 3 Results

### 3.1 Genetic Correlation Between Coffee Consumption and AMD

LDSC analysis results showed that the heritability (*h* ^2^) of coffee consumption, decaffeinated coffee consumption, ground coffee consumption, instant coffee consumption, and wet AMD, dry AMD were 0.110, 0.043, 0.059, 0.064, 0.026, and 0.025, respectively. There may not be a significant genetic correlation between coffee consumption, decaffeinated coffee consumption, and ground coffee consumption with dry AMD (Table 2). Instant coffee consumption was genetically correlated with dry AMD, and after correction using the BH method, the result remained significant (*p* = 0.045) (Table 2). A total of seven SNPs were selected for MR analysis between instant coffee and dry AMD, and we found that, after correction, there was no genetic correlation between wet AMD and instant coffee consumption (*p* = 0.124) (Table 2). To assess the potential sample overlap between the coffee consumption and AMD GWAS datasets, we examined the proportion of overlapping samples for each coffee consumption and AMD subtype. As shown in (Table 3), the overlap is minimal, with overlap percentages ranging from 0.4% to 1.2% across different coffee types and AMD phenotypes.

**TABLE 2:** Genetic correlations (*r*_g_) between coffee consumption traits and AMD subtypes, with FDR‐adjusted p‐values.

*r*_*g* (*p*adj)_	Dry AMD	Wet AMD
Coffee consumption	0. 03 (0.534)	0. 08 (0.246)
Decaffeinated coffee consumption	−0.02 (0.655)	0. 02 (0.722)
Ground coffee consumption	−0. 01 (0.751)	0. 08 (0.102)
Instant coffee consumption	0. 07 (0.045)	0. 06 (0.124)

Abbreviations: AMD, age‐related macular degeneration; *r*_*g*(*p*adj)_, genetic correlation between traits, and adjusted *p* values (*p*adj) using the Benjamini‐Hochberg method to control for FDR across multiple tests.

**TABLE 3:** Samples overlapping rate.

Samples overlapping rate	Dry AMD (%)	Wet AMD (%)
Coffee consumption	1.2	1.2
Decaffeinated coffee consumption	0.8	0.7
Ground coffee consumption	0.4	0.4
Instant coffee consumption	0.5	0.4

Abbreviation: AMD, age‐related macular degeneration.

### 3.2 UVMR Analysis

Using MR, as described earlier, we investigated the causal association between coffee consumption and AMD. The F‐statistics were all > 10, with the average *F*‐statistics for each exposure‐outcome pair ranging from 47.96 to 181.03 (Table 4). In addition, SNP‐specific *F*‐statistics were calculated (Table S2), confirming that all individual instrumental variables met the *F* > 10 criterion, thereby minimizing the risk of weak instrument bias. Sensitivity analyses using MR‐Egger and MR‐PRESSO further supported the robustness of our findings. IVW results showed that only instant coffee consumption had a statistically significant association with dry AMD after BH correction (*p* = 0.006) (Table 4). Results from all analysis methods are provided in Table S1. Genetically predicted instant coffee consumption was found to increase the risk of dry AMD, with each SD increase in instant coffee consumption corresponding to an OR of approximately 6.92 for dry AMD, indicating a 6.92‐fold increased risk. The individual SNPs used in UVMR analysis are shown in Table S2.

**TABLE 4:** The result of MR analysis.

Exposure	Outcome	Method	No. of SNP	BETA	SE	*p*	*p*_adj_	OR (95% CI)	*F* statistic
Coffee	DRY AMD	IVW	9	0.14	0.13	0.281	0.773	1.15 (0.89, 1.48)	125.72
Coffee	WET AMD	IVW	9	−0.05	0.14	0.698	0.920	0.95 (0.72, 1.25)	125.72
Decaffeinated Coffee	DRY AMD	IVW	2	−0.47	1.52	0.759	0.920	0.63 (0.03, 12.30)	47.96
Decaffeinated Coffee	WET AMD	IVW	2	−0.82	3.31	0.805	0.920	0.44 (0.00, 289.34)	47.96
Ground coffee	DRY AMD	IVW	2	0.59	1.85	0.752	0.920	1.80 (0.05, 67.76)	181.03
Ground coffee	WET AMD	IVW	2	−0.09	2.37	0.969	0.969	0.91 (0.01, 95.13)	180.89
Instant coffee	DRY AMD	IVW	7	2.07	0.76	0.006	0.048	7.92 (1.79, 35.15)	99.40
Instant coffee	WET AMD	IVW	7	0.95	0.9	0.29	0.773	2.59 (0.44, 15.09)	102.21

Abbreviations: BETA, effect size estimate; CI, confidence interval; *F* statistic, instrument strength statistic; OR, odds ratio; *p*, *p* value; *p*adj, adjusted *p* value; SE, standard error; SNP, single nucleotide polymorphism.

In sensitivity analysis, Cochran's Q test indicated the presence of heterogeneity in the MR analysis of ground coffee with dry and wet AMD, as well as between decaffeinated coffee and wet AMD. However, the WM analysis still suggested no potential causal association. Additionally, no heterogeneity was found between instant coffee and dry AMD (Table S3). The intercept test did not indicate the presence of pleiotropy in the previous MR analysis (Table S3).

### 3.3 Colocalization Analysis

To examine whether the exposure and outcome shared causal variants in the same test region, we applied the coloc method for colocalization analysis. First, we obtained independently associated SNPs (Table S4) from different coffee consumption and AMD categories, which were considered lead SNPs. These were then combined, and regions within a 500 KB upstream and downstream window were selected as test regions for analysis. Colocalization analysis results showed that there were no shared regions or genetic variants between coffee consumption and AMD (all PPH4 > 0.75) (Table S4).

## 4 Discussion

This study comprehensively investigated the risk of coffee consumption on AMD using large‐scale GWAS summary data. We analyzed the genetic correlation, causal association, and shared causal variants between coffee consumption and AMD. The results revealed a genetic link between coffee consumption and AMD, especially showing that instant coffee increases the risk of dry AMD.

Previous cohort studies suggested that coffee was beneficial for AMD (Chiu et al. 2017). In contrast, our study provided a more detailed stratification of coffee types and yielded different results, indicating potential biases in previous research. The LDSC results suggest a potential genetic correlation between instant coffee consumption and both dry and wet AMD. UVMR analysis results indicate that overall coffee consumption may not have a causal relationship with AMD. Interestingly, we found that instant coffee significantly increases the risk of AMD, with each SD increase in instant coffee consumption corresponding to an OR of approximately 6.92 for dry AMD, indicating a 6.92‐fold increased risk. This comprehensive analysis using various methods confirmed the reliable genetic link between coffee consumption and AMD.

The relationship between coffee and health has been well‐studied, showing both beneficial and detrimental effects. Previous reports suggested that coffee could improve liver steatosis and fibrosis and reduce the risk of these conditions (Morisco et al. 2014). Additionally, some studies indicated that decaffeinated coffee, ground coffee, and instant coffee might protect cardiovascular health, reducing the risk of coronary heart disease, and that excessive consumption is not associated with cardiovascular disease (O'Keefe et al. 2013; Ding et al. 2014; Chieng et al. 2022). Most research points to the protective effects of coffee. However, coffee consumption, especially, has been linked to an increased risk of ocular diseases. Specifically, coffee not only increases the risk of primary open‐angle glaucoma but also raises the risk of age‐related cataracts (Li et al. 2022; Yuan et al. 2022). Interestingly, coffee seems to have a significant impact on age‐related diseases. Although coffee consumption is positively correlated with longevity, instant coffee consumption is negatively correlated with the aging biomarker telomere length (Wei et al. 2023; O'Keefe et al. 2018). Similarly, we found no causal relationship between coffee and AMD, but a potential genetic and causal link between instant coffee and AMD. We speculate that the different effects of various types of coffee on AMD may be related to manufacturing processes, additives, and other factors (Moraes and Bolini 2010; Farah and Farah 2019; Neoh et al. 2016). For instance, studies have reported that the production of instant coffee may lead to the formation of potentially harmful substances such as acrylamide and advanced glycation end products (AGEs), which have been implicated in oxidative stress and inflammatory responses in retinal cells. Instant coffee is made by brewing a concentrated extract and then spray‐drying or freeze‐drying the liquid; this intense heat and concentration generates high levels of Maillard reaction byproducts and often involves added ingredients (e.g., sugar, creamer). Other types of coffee do not have similar additives. The production process also produces relatively few AGEs. Surveys of instant coffee chemistry show markedly elevated “neo‐formed” toxins (Zhang et al. 2023; Moore et al. 2003). AGEs activate multiple signaling pathways, including nuclear factor‐κB (NF‐κB), transforming growth factor‐β (TGF‐β), Jak–STAT, and PI3K‐Akt pathways, by binding to their receptor RAGE, leading to pathological changes such as inflammation, apoptosis, and angiogenesis. In addition, the accumulation of AGEs induces the production of reactive oxygen species (ROS), which disrupts the redox balance of retinal cells and further exacerbates oxidative stress and inflammatory responses. AGEs also increase the DNA‐binding activity of NF‐κB and the expression of intercellular adhesion molecule 1 (ICAM‐1) by interacting with RAGE, which promotes leukocyte adhesion to retinal endothelial cells, leading to disruption of the blood‐retinal barrier (Kang et al. 2022).

The current study showed that co‐localization analyses did not identify shared genetic regions or variants between coffee intake and AMD, suggesting that the causal effect of instant coffee on the risk of dry AMD may not be driven by a single genetic variant, but rather involves a variety of complex biological mechanisms, including polygenic regulatory effects, gene–environment interactions, and epigenetic modifications. Although significant MR analyses supported a causal relationship between instant coffee and dry AMD, the effect could not be clearly attributed to a single variant in co‐localization analyses, leading to the hypothesis that instant coffee may comprehensively regulate biological processes associated with AMD through multiple genetic factors interacting with the environment and influencing epigenetic modifications. In addition, the effects of instant coffee may also be partially attributable to inflammation and oxidative stress induced by the harmful chemicals produced during its production. Future studies need to synthesize these complex mechanisms from multiple perspectives to further reveal the intrinsic link between instant coffee and dry AMD, and provide a more comprehensive theoretical basis for disease prevention and intervention.

This study provides guidance regarding instant coffee consumption, suggesting that it increases the risk of dry AMD, and therefore high‐risk groups should reduce their intake. Furthermore, it is essential to identify harmful components in instant coffee and other types of coffee to reduce their content. Exploring the mechanisms of instant coffee's effects on AMD using animal models is also necessary. Additionally, further analysis of the genetic associations between instant coffee and other diseases is needed to understand the broader impacts of coffee on health.

## 5 Strengths and Limitations

We combined genetic correlation analysis and causal inference to investigate the genetic link between coffee consumption and AMD, which allows for mutual supplementation to avoid biases caused by using a single method. Furthermore, we utilized the latest AMD data, providing a more accurate and objective analysis of the relationship between coffee consumption and AMD.

However, our study has some limitations. Firstly, although there was some sample overlap which was very low and we employed multiple methods to address it, potential bias cannot be completely ruled out. Secondly, due to the lack of individual data, we did not conduct nonlinear MR analysis on the relationship between coffee consumption and AMD risk. Thirdly, our analysis was limited to the European population, and future studies should consider including more diverse populations. Finally, our classification of coffee consumption was not detailed enough, and future studies should consider more refined classifications, such as “dark roast coffee” or “medium roast coffee,” which may uncover more potential risks associated with AMD.

## 6 Conclusion

Instant coffee may increase the risk of AMD, and reducing instant coffee intake can prevent dry AMD. People at high‐risk for AMD should avoid instant coffee intake.

## Author Contributions

**Qi Jia:** writing – original draft (equal). **Zhijian Zha:** writing – original draft (equal). **Si Li:** software (equal). **Yong Zhang:** software (equal), writing – review and editing (equal). **Lan Ke:** data curation (equal), writing – review and editing (equal). **Siwei Liu:** funding acquisition (equal).

## Disclosure

Human Ethics and Consent to Participate Declarations: Not applicable.

## Conflicts of Interest

The authors declare no conflicts of interest.

## Supporting information


**Table S1.** All result of MR analysis.
**Table S2.** The single SNPs for MR Analysis.
**Table S3.** The result of sensitivity test.
**Table S4.** The result of colocalization.

## Data Availability

All GWAS summary data sources have been cited within the manuscript. The code used for the analyses is available as follows: LDSC (v 1.0.1) for analyzing heritability and genetic correlations between traits, with code from https://github.com/bulik/ldsc. The MR analysis was conducted using the TwoSampleMR (version 0.6.6) R package, with code available at https://github.com/MRCIEU/TwoSampleMR. The analysis code for BVMR and RAPS can be found at https://github.com/RealMrCactus/BVMR/ and https://github.com/mihdalal/raps, respectively.

## References

[fsn370439-bib-0001] Auton, A. , L. D. Brooks , R. M. Durbin , et al. 2015. “A Global Reference for Human Genetic Variation.” Nature 526: 68–74. 26432245 10.1038/nature15393 PMC4750478

[fsn370439-bib-0002] Birney, E. 2022. “Mendelian Randomization.” Cold Spring Harbor Perspectives in Medicine 12: a041302. 34872952 10.1101/cshperspect.a041302 PMC9121891

[fsn370439-bib-0003] Bowden, J. , G. Davey Smith , P. C. Haycock , and S. Burgess . 2016. “Consistent Estimation in Mendelian Randomization With Some Invalid Instruments Using a Weighted Median Estimator.” Genetic Epidemiology 40: 304–314. 27061298 10.1002/gepi.21965 PMC4849733

[fsn370439-bib-0004] Bulik‐Sullivan, B. , H. K. Finucane , V. Anttila , et al. 2015. “An Atlas of Genetic Correlations Across Human Diseases and Traits.” Nature Genetics 47: 1236–1241. 26414676 10.1038/ng.3406 PMC4797329

[fsn370439-bib-0005] Burgess, S. , F. Dudbridge , and S. G. Thompson . 2016. “Combining Information on Multiple Instrumental Variables in Mendelian Randomization: Comparison of Allele Score and Summarized Data Methods.” Statistics in Medicine 35: 1880–1906. 26661904 10.1002/sim.6835 PMC4832315

[fsn370439-bib-0006] Burgess, S. , D. S. Small , and S. G. Thompson . 2017. “A Review of Instrumental Variable Estimators for Mendelian Randomization.” Statistical Methods in Medical Research 26: 2333–2355. 26282889 10.1177/0962280215597579 PMC5642006

[fsn370439-bib-0007] Burgess, S. , and S. G. Thompson . 2011. “Avoiding Bias From Weak Instruments in Mendelian Randomization Studies.” International Journal of Epidemiology 40: 755–764. 21414999 10.1093/ije/dyr036

[fsn370439-bib-0008] Chieng, D. , R. Canovas , L. Segan , et al. 2022. “The Impact of Coffee Subtypes on Incident Cardiovascular Disease, Arrhythmias, and Mortality: Long‐Term Outcomes From the UK Biobank.” European Journal of Preventive Cardiology 29: 2240–2249. 36162818 10.1093/eurjpc/zwac189

[fsn370439-bib-0009] Chiu, C. J. , M. L. Chang , T. Li , G. Gensler , and A. Taylor . 2017. “Visualization of Dietary Patterns and Their Associations With Age‐Related Macular Degeneration.” Investigative Ophthalmology & Visual Science 58: 1404–1410. 28253403 10.1167/iovs.16-20454 PMC5361454

[fsn370439-bib-0010] Deng, Y. , L. Qiao , M. Du , et al. 2022. “Age‐Related Macular Degeneration: Epidemiology, Genetics, Pathophysiology, Diagnosis, and Targeted Therapy.” Genes & Diseases 9: 62–79. 35005108 10.1016/j.gendis.2021.02.009 PMC8720701

[fsn370439-bib-0011] Ding, M. , S. N. Bhupathiraju , A. Satija , R. M. van Dam , and F. B. Hu . 2014. “Long‐Term Coffee Consumption and Risk of Cardiovascular Disease: A Systematic Review and a Dose‐Response Meta‐Analysis of Prospective Cohort Studies.” Circulation 129: 643–659. 24201300 10.1161/CIRCULATIONAHA.113.005925 PMC3945962

[fsn370439-bib-0012] Farah, A. , and A. Farah . 2019. Coffee: Production, Quality and Chemistry. Royal Society of Chemistry.

[fsn370439-bib-0013] Fleckenstein, M. , S. Schmitz‐Valckenberg , and U. Chakravarthy . 2024. “Age‐Related Macular Degeneration: A Review.” JAMA 331: 147–157. 38193957 10.1001/jama.2023.26074 PMC12935482

[fsn370439-bib-0014] Fritsche, L. G. , R. N. Fariss , D. Stambolian , G. R. Abecasis , C. A. Curcio , and A. Swaroop . 2014. “Age‐Related Macular Degeneration: Genetics and Biology Coming Together.” Annual Review of Genomics and Human Genetics 15: 151–171. 10.1146/annurev-genom-090413-025610 PMC4217162 24773320

[fsn370439-bib-0015] Giambartolomei, C. , J. Zhenli Liu , W. Zhang , et al. 2018. “A Bayesian Framework for Multiple Trait Colocalization From Summary Association Statistics.” Bioinformatics (Oxford, England) 34: 2538–2545. 29579179 10.1093/bioinformatics/bty147 PMC6061859

[fsn370439-bib-0016] Kang, Q. , H. Dai , S. Jiang , and L. Yu . 2022. “Advanced Glycation End Products in Diabetic Retinopathy and Phytochemical Therapy.” Frontiers in Nutrition 9: 1037186. 10.3389/fnut.2022.1037186. 36466410 PMC9716030

[fsn370439-bib-0017] Kim, J. , H. Aschard , J. H. Kang , et al. 2021. “Intraocular Pressure, Glaucoma, and Dietary Caffeine Consumption: A Gene‐Diet Interaction Study From the UK Biobank.” Ophthalmology 128: 866–876. 33333105 10.1016/j.ophtha.2020.12.009 PMC8154631

[fsn370439-bib-0018] Klein, R. J. , C. Zeiss , E. Y. Chew , et al. 2005. “Complement Factor H Polymorphism in Age‐Related Macular Degeneration.” Science (New York, N.Y.) 308: 385–389. 15761122 10.1126/science.1109557 PMC1512523

[fsn370439-bib-0019] Kurki, M. I. , J. Karjalainen , P. Palta , et al. 2023. “FinnGen Provides Genetic Insights From a Well‐Phenotyped Isolated Population.” Nature 613: 508–518. 36653562 10.1038/s41586-022-05473-8 PMC9849126

[fsn370439-bib-0020] Li, X. , S. Cheng , J. Cheng , M. Wang , Y. Zhong , and A. Y. Yu . 2022. “Habitual Coffee Consumption Increases Risk of Primary Open‐Angle Glaucoma: A Mendelian Randomization Study.” Ophthalmology 129: 1014–1021. 35537532 10.1016/j.ophtha.2022.04.027

[fsn370439-bib-0021] Liu, D. , M. Cao , H. Wang , et al. 2024. “Association Between Inflammatory Bowel Disease and Cancer Risk: Evidence Triangulation From Genetic Correlation, Mendelian Randomization, and Colocalization Analyses Across East Asian and European Populations.” BMC Medicine 22: 137. 38528540 10.1186/s12916-024-03352-9 PMC10964701

[fsn370439-bib-0022] Moore, T. C. B. , J. E. Moore , Y. Kaji , et al. 2003. “The Role of Advanced Glycation End Products in Retinal Microvascular Leukostasis.” Investigative Ophthalmology & Visual Science 44: 4457–4464. 10.1167/iovs.02-1063. 14507893

[fsn370439-bib-0023] Moraes, P. , and H. Bolini . 2010. “Different Sweeteners in Beverages Prepared With Instant and Roasted Ground Coffee: Ideal and Equivalent Sweetness.” Journal of Sensory Studies 25: 215–225.

[fsn370439-bib-0024] Morisco, F. , V. Lembo , G. Mazzone , S. Camera , and N. Caporaso . 2014. “Coffee and Liver Health.” Journal of Clinical Gastroenterology 48, no. Suppl 1: S87–S90. 25291138 10.1097/MCG.0000000000000240

[fsn370439-bib-0025] Neoh, T. L. , S. Adachi , and T. Furuta . 2016. “Instant Coffee.” In Introduction to Food Manufacturing Engineering, 65–89. Springer Singapore.

[fsn370439-bib-0026] O'Keefe, J. H. , S. K. Bhatti , H. R. Patil , J. J. DiNicolantonio , S. C. Lucan , and C. J. Lavie . 2013. “Effects of Habitual Coffee Consumption on Cardiometabolic Disease, Cardiovascular Health, and All‐Cause Mortality.” Journal of the American College of Cardiology 62: 1043–1051. 23871889 10.1016/j.jacc.2013.06.035

[fsn370439-bib-0027] O'Keefe, J. H. , J. J. DiNicolantonio , and C. J. Lavie . 2018. “Coffee for Cardioprotection and Longevity.” Progress in Cardiovascular Diseases 61: 38–42. 29474816 10.1016/j.pcad.2018.02.002

[fsn370439-bib-0028] Pirastu, N. , C. McDonnell , E. J. Grzeszkowiak , et al. 2022. “Using Genetic Variation to Disentangle the Complex Relationship Between Food Intake and Health Outcomes.” PLoS Genetics 18: e1010162. 35653391 10.1371/journal.pgen.1010162 PMC9162356

[fsn370439-bib-0029] Rasooly, D. , G. M. Peloso , and C. Giambartolomei . 2022. “Bayesian Genetic Colocalization Test of Two Traits Using Coloc.” Current Protocols 2: e627. 36515558 10.1002/cpz1.627

[fsn370439-bib-0030] Sanderson, E. , M. M. Glymour , M. V. Holmes , et al. 2022. “Mendelian Randomization.” Nature Reviews Methods Primers 2: 6. 10.1038/s43586-021-00092-5 PMC7614635 37325194

[fsn370439-bib-0031] Sekula, P. , M. F. Del Greco , C. Pattaro , and A. Köttgen . 2016. “Mendelian Randomization as an Approach to Assess Causality Using Observational Data.” Journal of the American Society of Nephrology 27: 3253–3265. 27486138 10.1681/ASN.2016010098 PMC5084898

[fsn370439-bib-0032] Song, J. W. , and K. C. Chung . 2010. “Observational Studies: Cohort and Case‐Control Studies.” Plastic and Reconstructive Surgery 126: 2234–2242. 20697313 10.1097/PRS.0b013e3181f44abc PMC2998589

[fsn370439-bib-0033] Stahl, A. 2020. “The Diagnosis and Treatment of Age‐Related Macular Degeneration.” Deutsches Ärzteblatt International 117: 513–520. 33087239 10.3238/arztebl.2020.0513 PMC7588619

[fsn370439-bib-0034] van Lookeren Campagne, M. , J. LeCouter , B. L. Yaspan , and W. Ye . 2014. “Mechanisms of Age‐Related Macular Degeneration and Therapeutic Opportunities.” Journal of Pathology 232: 151–164. 24105633 10.1002/path.4266

[fsn370439-bib-0035] Vassy, J. L. , Y. L. Ho , J. Honerlaw , et al. 2018. “Yield and Bias in Defining a Cohort Study Baseline From Electronic Health Record Data.” Journal of Biomedical Informatics 78: 54–59. 29305952 10.1016/j.jbi.2017.12.017 PMC5846098

[fsn370439-bib-0036] Wei, Y. , Z. Li , H. Lai , et al. 2023. “Instant Coffee Is Negatively Associated With Telomere Length: Finding From Observational and Mendelian Randomization Analyses of UK Biobank.” Nutrients 15: 1354. 36986083 10.3390/nu15061354 PMC10055626

[fsn370439-bib-0037] Xiao, G. , Q. He , L. Liu , et al. 2022. “Causality of Genetically Determined Metabolites on Anxiety Disorders: A Two‐Sample Mendelian Randomization Study.” Journal of Translational Medicine 20: 475. 36266699 10.1186/s12967-022-03691-2 PMC9583573

[fsn370439-bib-0038] Yuan, S. , A. Wolk , and S. C. Larsson . 2022. “Metabolic and Lifestyle Factors in Relation to Senile Cataract: A Mendelian Randomization Study.” Scientific Reports 12: 409. 35013517 10.1038/s41598-021-04515-x PMC8748724

[fsn370439-bib-0039] Zhang, F. , H. Liu , and Y. Wang . 2023. “Production and Inhibition of Acrylamide During Coffee Processing.” Food Science & Nutrition 11: 1305–1313. 10.1016/j.foodchem.2023.1305.

[fsn370439-bib-0040] Zhang, Y. , Y. Cheng , W. Jiang , Y. Ye , Q. Lu , and H. Zhao . 2021. “Comparison of Methods for Estimating Genetic Correlation Between Complex Traits Using GWAS Summary Statistics.” Briefings in Bioinformatics 22: bbaa442. 33497438 10.1093/bib/bbaa442 PMC8425307

[fsn370439-bib-0041] Zhao, J. , J. Ming , X. Hu , G. Chen , J. Liu , and C. Yang . 2020. “Bayesian Weighted Mendelian Randomization for Causal Inference Based on Summary Statistics.” Bioinformatics (Oxford, England) 36: 1501–1508. 31593215 10.1093/bioinformatics/btz749

[fsn370439-bib-0042] Zhao, Q. , J. Wang , G. Hemani , J. Bowden , and D. S. Small . 2020. “Statistical Inference in Two‐Sample Summary‐Data Mendelian Randomization Using Robust Adjusted Profile Score.” Annals of Statistics 48: 1742–1769.

